# The effect of a barrier membrane on the incorporation of deproteinized bovine bone mineral (DBBM) in experimental defects at the time of early implant placement. A preclinical study

**DOI:** 10.1007/s00784-024-05748-6

**Published:** 2024-06-01

**Authors:** Rebecca Ellis, Stephen Chen, Fernando Mª Muñoz Guzón, Ivan Darby

**Affiliations:** 1 Melbourne Dental School, University of Melbourne, Parkville, VIC Australia; 2 Facultade de Veterinaria, Campus Universitario, Lugo, Spain; 3 Melbourne Dental School, University of Melbourne, 720 Swanston Street, Carlton, VIC 3053 Australia

**Keywords:** Alveolar ridge defect, Bone graft, Bone substitute, Dental implants, Guided bone regeneration, Membrane, Cattle, Animals, Dogs, Bone substitutes, Incisor, Bone regeneration, Minerals, Collagen

## Abstract

**Objectives:**

This study aimed to assess membrane use with a bone substitute graft for guided bone regeneration (GBR) in experimental dehiscence defects.

**Materials and methods:**

Maxillary second incisors (I2) in 9 dogs were extracted. Six weeks later, implants were inserted and experimental dehiscence defects (5 × 3 mm) created on the buccal aspect. The defects and surrounding bone were grafted with deproteinized bovine bone mineral. One side (test) was covered with a resorbable collagen membrane whereas the contralateral side (control) was not. After 6 weeks, histomorphometrical analysis was performed to evaluate: (a) first bone-to-implant contact (fBIC), (b) buccal bone thickness at 1 mm increments from implant shoulder, (c) regenerated area (RA), (d) area and percentages of new bone (B), bone substitute (BS) and mineralized tissue (MT).

**Results:**

The histological appearance was similar between test and control sites. At central and lateral sections, there were no differences between groups for fBIC, buccal bone thickness, RA, BS, B, %B, MT and %MT. At central sections, membrane use favoured more %BS and %MT (*p* = 0.052). There was significantly more B, %B and MT at lateral compared to central sections.

**Conclusions:**

Membrane use tended to retain more bone substitute, but had no effect on new bone ingrowth. Lateral sections showed significantly more bone ingrowth and mineralized tissue compared to central sections, confirming that new bone ingrowth takes place mainly from the lateral walls of the defect.

**Clinical relevance:**

Preclinical research to clarify the dynamics of bone regeneration in GBR procedures is relevant in clinical practice.

## Introduction

Guided bone regeneration (GBR) has been defined as a technique to maintain a space at a bony defect by using a barrier membrane, thereby excluding epithelial cells and fibroblasts, and permitting ingrowth of osteogenic cells and blood vessels [1]. Bone grafts and bone substitutes are often used in conjunction with the GBR procedure to provide physical support to the membrane and overlying soft tissues, and to prevent collapse of the membrane into the defect [2]. Resorbable xenogeneic collagen membranes and deproteinized bovine bone mineral (DBBM) are the predominant materials used for this purpose [3]. Initial pre-clinical studies investigating the efficacy of barrier membranes with DBBM grafts focused on experimental bone defects, and reported contrasting results. In circumferential defects in porcine and rodent models, more bone regeneration was observed at the centre region and at larger defects when membranes were used [4, 5]. In contrast, a study of similar defects created in the mandible of dogs showed no difference whether a membrane was used in conjunction with DBBM or not [6]. In a study of experimental buccal dehiscence defects in the dog mandible, the use of a membrane in conjunction with DBBM resulted in greater radiographic bone density within the grafted defect when a membrane was used compared to no membrane [7].

The efficacy of barrier membranes and DBBM grafts have also been evaluated in experimental peri-implant defects. In a study of circumferential defects at dental implants grafted with DBBM and protected with a membrane, similar percentage new bone formation was observed whether a membrane was used or not [8]. A more challenging peri-implant defect is the buccal dehiscence defect. This defect type is often encountered clinically with early post-extraction implant placement [9], and is commonly managed with the GBR procedure utilizing a resorbable collagen membrane and DBBM [2]. In aesthetic sites, the DBBM material is also placed in excess on the buccal aspect to augment the contour of the ridge. There have been few preclinical studies examining the histological outcomes when managing peri-implant dehiscence defects with a barrier membrane and DBBM [10, 11]. In a study of dehiscence defects on the buccal aspect of implants in the mandible of dogs, sites grafted with DBBM were compared to sites grafted with DBBM + collagen membrane. Significantly greater regenerated area was noted at sites receiving a membrane [11]. In contrast, a study of dehiscence defects on the buccal aspect of implants placed in the maxillary second incisor and first premolar sites showed no difference in the regenerated area whether a membrane was used or not [10]. The results of these studies suggest that barrier membranes may not enhance the volume of the grafted area, but may increase the percentage of new bone forming within the defect. There a few studies investigating the regeneration in the anterior maxilla which is a common clinical procedure. The majority of studies have been undertaken in the posterior mandible which not reflective of the anterior maxilla. The maxillary second incisor model recently introduced is a unique model as it allows studies on immediate and early implant placement in single tooth maxillary sites with adjacent natural teeth, thereby more closely resembling the clinical situation and therefore providing additional evidence to support translational interpretation. In addition, the second maxillary incisor is a similar size to human teeth. Further pre-clinical studies are required to verify if barrier membranes affect the volume of grafted bone in the anterior maxilla.

Therefore, the aim of the present study was to investigate the efficacy of membrane use in GBR procedures by histologically and histomorphometrically evaluating healing of DBBM grafted experimental dehiscence bone defects with or without membrane following early (type 2) implant placement in second maxillary incisor sites in the canine model.

## Materials and methods

This study was designed as a randomized experimental study. The Ethical Committee of the Rof Codina Foundation (Lugo, Spain) approved the study protocol (01/17/LU-001). The animals were housed in the animal experimental service facility of the Rof Codina Foundation (Cebiovet, Faculty of Veterinary Medicine, Lugo, Spain). All the experiments were performed according to Spanish and European regulations about care and use of research animals, in full compliance with the ARRIVE guidelines [12].

### Animals

A total of nine healthy adult female Mongrel Hound type dogs, mean age 16.5 (±0.5) months, weight 19–22 kg (Mean 20.5 kg) (Marshall Bioresources, North Rose, NY, USA) were used in this experimental in vivo investigation.

Sample size was calculated using SigmaPlot 12.5 software (Systat Software Inc., Chicago, IL, USA). The outcome of this calculation was eight, which using a split mouth design resulted in 8 dogs. Researchers decided to include a ninth dog to avoid lack of statistical power in case of an unexpected loss during the experimental phase.

Animals were maintained in a group kennel with indoor and outdoor areas and were fed using granulated dog food, previously wetted in water, with individual bowls and free supply of water. Dogs were monitored daily throughout the study period by a veterinarian accredited in laboratory animal science. The experimental segment of the study started after a quarantine period of 3 weeks.

### Surgical procedures

All surgical procedures were done under sterile conditions, in an animal operating theatre and under general anaesthesia induced by propofol (3–5 mg/ kg/i.v., Propovet®, Abbott Laboratories, Kent, UK), and maintained on a concentration of 2.5–4% of isoflurane (Isoba-vet®, Schering-Plough, Madrid, Spain). The animals were first premedicated with medetomidine (20 μg/kg/i.m., Domtor, Esteve, Barcelona, Spain) and the pain controlled with the administration of morphine (0.4 mg/kg/i.m., Morfina Braun 2%, B. Braun Medical, Barcelona, Spain). Continuous monitoring using electrocardiography, capnography and pulse oximetry, and non-invasive blood pressure assessment was performed continuously during anaesthesia by a veterinarian. At the end of the procedure Atipamezol (50 μg/kg/i.m., Esteve, Barcelona, Spain) was administered to revert the effects of the Medetomidine. Postoperative pain was controlled by administration of morphine (0.2 mg/kg/i.m./6 h, Morfina Braun 2%, B. Braun Medical, Barcelona, Spain) and meloxicam as anti-inflammatory and analgesic treatment (0.2 mg/kg/i.m./SID, Metacam, Boehringer Ingelheim, Barcelona, Spain) 5 days postoperatively. Prophylactic administration of cefazolin (20 mg/kg/s.c./SID, Kurgan; Normon, Spain) and cefovecin (8 mg/kg/s.c./SID, Convenia; Zoetis, Spain) was performed intraoperatively. The oral mucosa, teeth and implants were disinfected three times a week by using gauzes soaked in a 0.12% chlorhexidine solution (Perio-Aid Tratamiento, Dentaid, Barcelona, Spain) for the first two weeks. Subsequently, a toothbrush and a chlorhexidine (0.2%) gel were used for plaque control until euthanasia.

#### Procedure #1

The second incisors in both quadrants of the maxilla (I2) were used as experimental sites. An intrasulcular incision was performed prior to extraction using elevators and forceps. Following successful delivery of the tooth, the corono-apical depth and bucco-lingual width of sockets as well as the thickness of crestal bone at the mid-buccal point were measured using a Michigan O probe with Williams markings (Hu-Friedy, Chicago, IL, USA) and recorded by rounding off the measurements to the nearest half a millimetre. Sockets were cleaned of any remnants of soft tissue and checked for any possible dehiscence and/or fenestration of the buccal bone. The sites were left to spontaneously heal.

#### Procedure #2

**Fig. 1:**
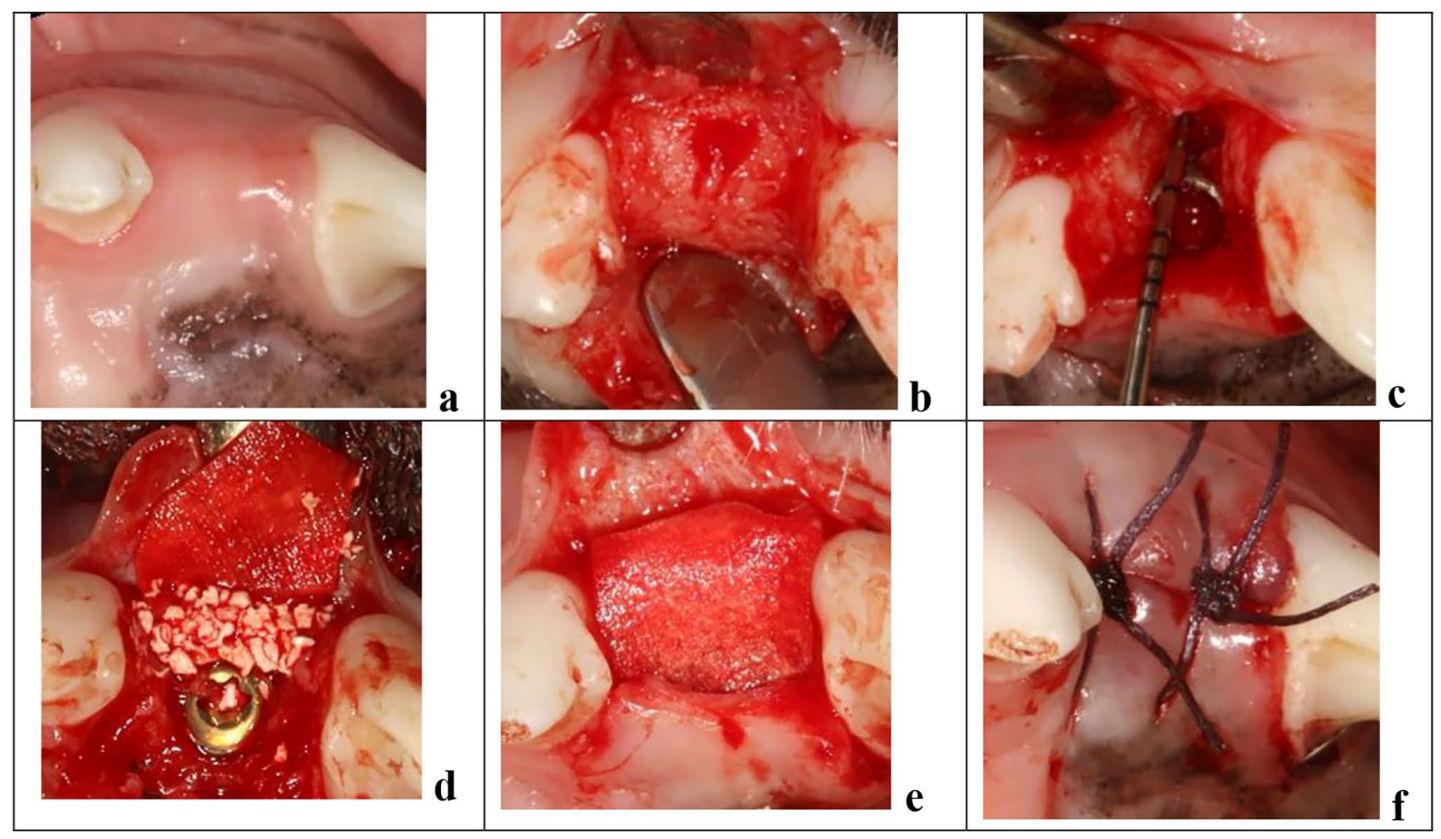
Surgical procedure at the time of implant placement. (**a**) Healed site 6 weeks after extraction of the maxillary second incisor (I2). (**b**) A full thickness mucoperiosteal flap was raised. (**c**) Following implant placement and creation of the experimental defect at the buccal aspect measuring 3 × 5 mm. (**d**) Grafting of the defect and buccal surface of the adjacent bone with deproteinized bovine bone mineral (DBBM). (**e**) A resorbable collagen membrane covered the graft. (**f**) Primary flap closure

Following a healing period of 6 weeks (Fig. 1a), a second surgical procedure was undertaken for implant placement, creation of the experimental defect and bone augmentation.

A mid-crestal incision with a surgical scalpel blade was performed at both edentulous maxillary second incisor sites; this was followed by intrasulcular incisions at the adjacent teeth and vertical releasing incisions bilaterally at disto-buccal and mesio-buccal line angles at both sites. A mucoperiosteal flap was raised to allow visualization of the buccal bone (Fig. 1b).

The osteotomy was initiated at the mid-crestal position and completed in accordance with the manufacturer’s recommendations (Straumann Dental Implant System, Straumann, Basel, Switzerland) to house an 8 mm long and 3.3 mm wide implant. A Straumann Roxolid^®^ narrow CrossFit (NC) bone level tapered (BLT) implant with SLA^®^ surface was installed into the prepared osteotomy with the shoulder of the implant flush with the buccal bone crest. A NC closure cap was connected to the implants.

A surgical defect was prepared at the buccal aspect of the implant using a 2.3 mm round bur (Straumann Dental Implant System, Straumann, Basel, Switzerland), measuring 3 mm in mesio-distal width and 5 mm in corona-apical height (Fig. 1c). The experimental defect was filled with deproteinized bovine bone mineral (DBBM) with a particle size of 0.5–1 mm, (Cerabone^®^Granulate, Mebios GmbH, Dieburg, Germany). Additional graft was placed over the defect and the adjacent bone walls to augment the contour of the ridge as described by Buser and co-workers [2] (Fig. 1d). Randomization was performed by tossing a coin for each dog to determine the side to receive the barrier membrane (test site). At test sites, a resorbable collagen membrane (Jason^®^membrane, Botiss Biomaterials GmbH, Zossen, Germany) was placed over the grafted area, buccal bone and over the shoulder of the implant (Fig. 1e). The control sites did not receive a membrane. Periosteal releasing was performed and the flaps coronally advanced to achieve tension-free primary closure and sutured using 4 − 0 synthetic bio-resorbable sutures (Vicryl 4 − 0, Ethicon, Johnson & Johnson Medical Pty Ltd, North Ryde, Australia) (Fig. 1f). An occlusal radiograph was taken to record the implant position.

### Retrieval of specimens and histological preparation

Six weeks following the second experimental procedure, all nine dogs were euthanized with an overdose of intravenous injection of sodium pentobarbital (40–60 mg/kg/i.v., Dolethal, Vetoquinol, France) following prior sedation with medetomidine (30 μg/kg/i.m., Esteve, Barcelona, Spain). Subsequently, the maxillae were dissected and fixed in buffered 10% formaldehyde solution at a temperature of 4ºC for a week.

### Histological examination

Blocks containing the implant and the tissues around were obtained using an oscillating saw. The blocks were dehydrated in different graded ethanol series, infiltrated with different graded mixtures of ethanol and resin (Technovit 7200 VLC, Heraus Kulzer, Werheim, Germany) [13] and polymerized.

Longitudinal sections of 200 μm in a buccolingual direction were obtained using a band saw and mechanically micropolished (Exakt Apparatebau, Norderstedt, Germany) using silicon carbide papers (Struers, Copenhagen, Denmark) until obtained samples were a thickness of approximately 40 μm. Sections at the mid-buccal (central) and 1 mm laterally to the central regions of the defects were obtained. The slides were stained using Levai-Laczkó method [14] and images captured using a motorized stage transmission light microscope and a PC-based capture system (BX51, DP71, Olympus Corporation, Japan).

### Histomorphometric analysis

**Fig. 2:**
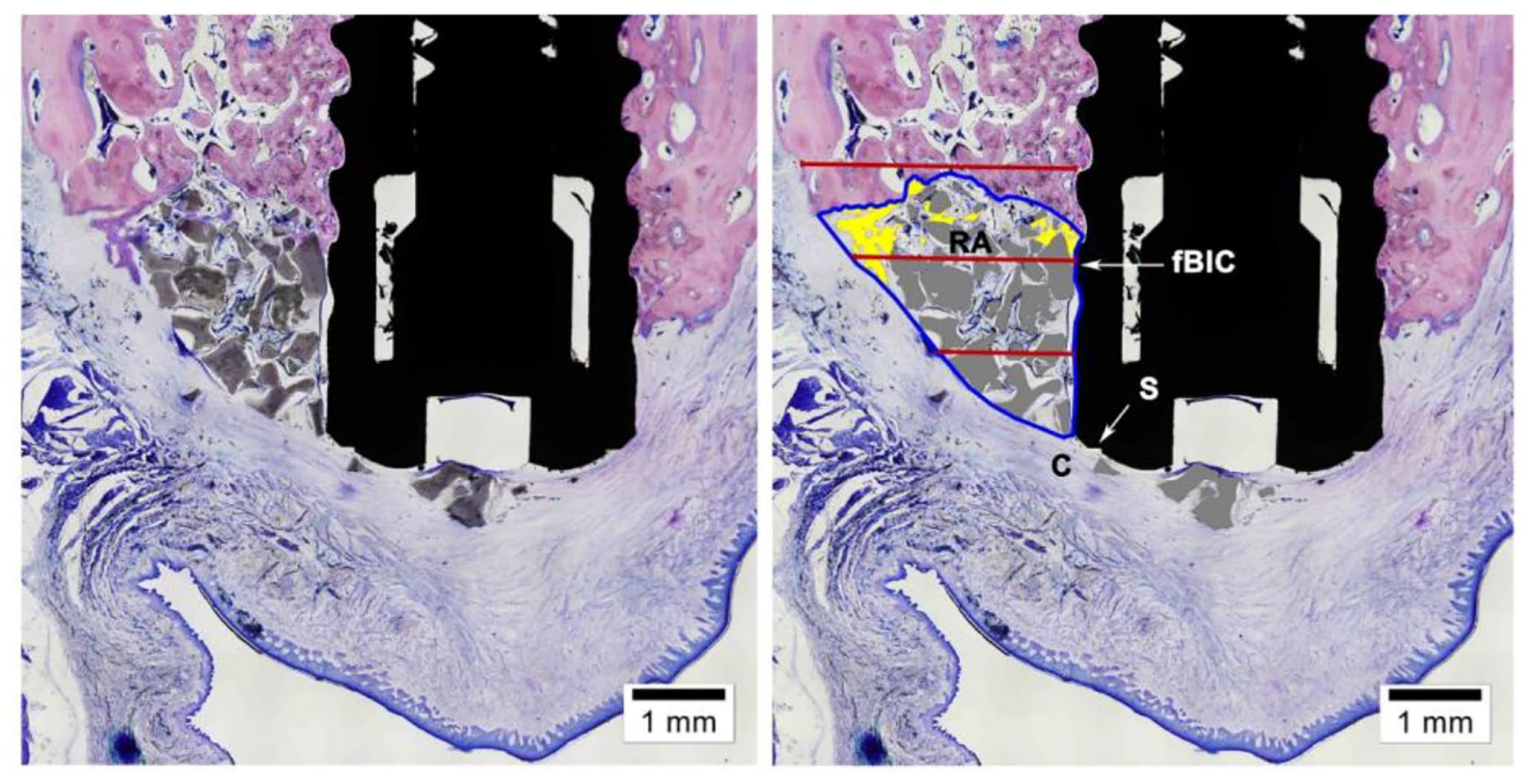
Schematic summary of the histomorphometric landmarks. Original image is on the left, computer enhanced image is on the right with landmarks. Buccal shoulder of the implant (S), first bone to implant contact (fBIC), crest of the buccal bone (C), and regenerated area (RA) shown within the blue outline. The width of the RA was measured at 1 mm increments from S (depicted in red horizontal lines. Bone substitute in the RA is coloured grey. New bone is coloured in yellow

Measurements were done using a PC-based image analysis program (Cell-sens 1.13, Olympus Corporation, Japan). All reference points in the histologic sections were marked by two experienced examiners independently and thereafter compared and discussed to aim for congruence. Measurements were then obtained by one examiner who was trained on the use of the software before initiating the measurements. The analysis included the following landmarks, distances and tissue components (Fig. 2):

- S: Shoulder of the implant (mm).
- C: the crest of the buccal bone wall (mm).
- fBIC: the most coronal point of contact between bone and implant (mm).
- S-fBIC: distance between the shoulder and the first bone-implant contact (mm).
- Widths of buccal bone wall at 1, 2, 3, 4 and 5 mm apical to S (mm).
- Regenerated area (RA) (mm^2^).
- Area and percentages of new bone (B (mm^2^) and %B), bone substitute (BS (mm^2^) and %BS) and mineralized tissue (MT (mm^2^) and %MT) within the RA.

### Data analysis

Summary statistics for the parameters of interest were prepared, and presented as the mean, standard deviation, median and interquartile range. The Wilcoxon signed rank test was used to analyse differences between the treatment groups for S-fBIC and buccal bone thickness at 1 mm increments from S (Minitab 20.2; Minitab Inc., State College, PA, USA). Two specimens (one test and one control) presented with significant bone resorption at the base of the experimental defects. The defects in these specimens were substantially different from the remaining specimens and were excluded in the subsequent analysis. This left seven paired specimens for analysis of RA, B, %B, BS, %BS, MT and %MT using the Wilcoxon signed rank test. The sections mesial and distal to the central section were analysed separately and found to be no different between groups. The data was then pooled was analysis. For comparisons between central and lateral sections, the data for membrane and no membrane groups were combined. The level of significance was set at 0.05.

## Results

### Clinical observations

Following extraction, one out of 18 sockets demonstrated a buccal bone dehiscence. There were no statistically significant differences in corono-apical depth and bucco-palatal width of the sockets between groups. Similarly, the thickness of the buccal bone was similar between groups. The mean combined corono-apical depth and bucco-palatal width of the sockets was 11.3 ± 1.1 mm (median 12 mm; range 9–13 mm) and 5.1 ± 0.7 mm (median 5 mm; range 4–6 mm). The mean combined thickness of the buccal crestal bone was 0.6 ± 0.1 mm (median 0.5 mm; range 0.5–1 mm).

After 6-weeks of healing, all sites demonstrated complete soft tissue closure. Upon reflection of the buccal flaps, the buccal bone was observed to be intact at all sites with absence of fenestration or dehiscence defects, including the one site with a buccal dehiscence at the time of extraction.

Following implant placement, defect creation and grafting, all sites healed uneventfully. At the end of the experimental period, all implants were submerged beneath the mucosa and were observed to be free of inflammation or other biological complications.

### Descriptive histology

**Fig. 3:**
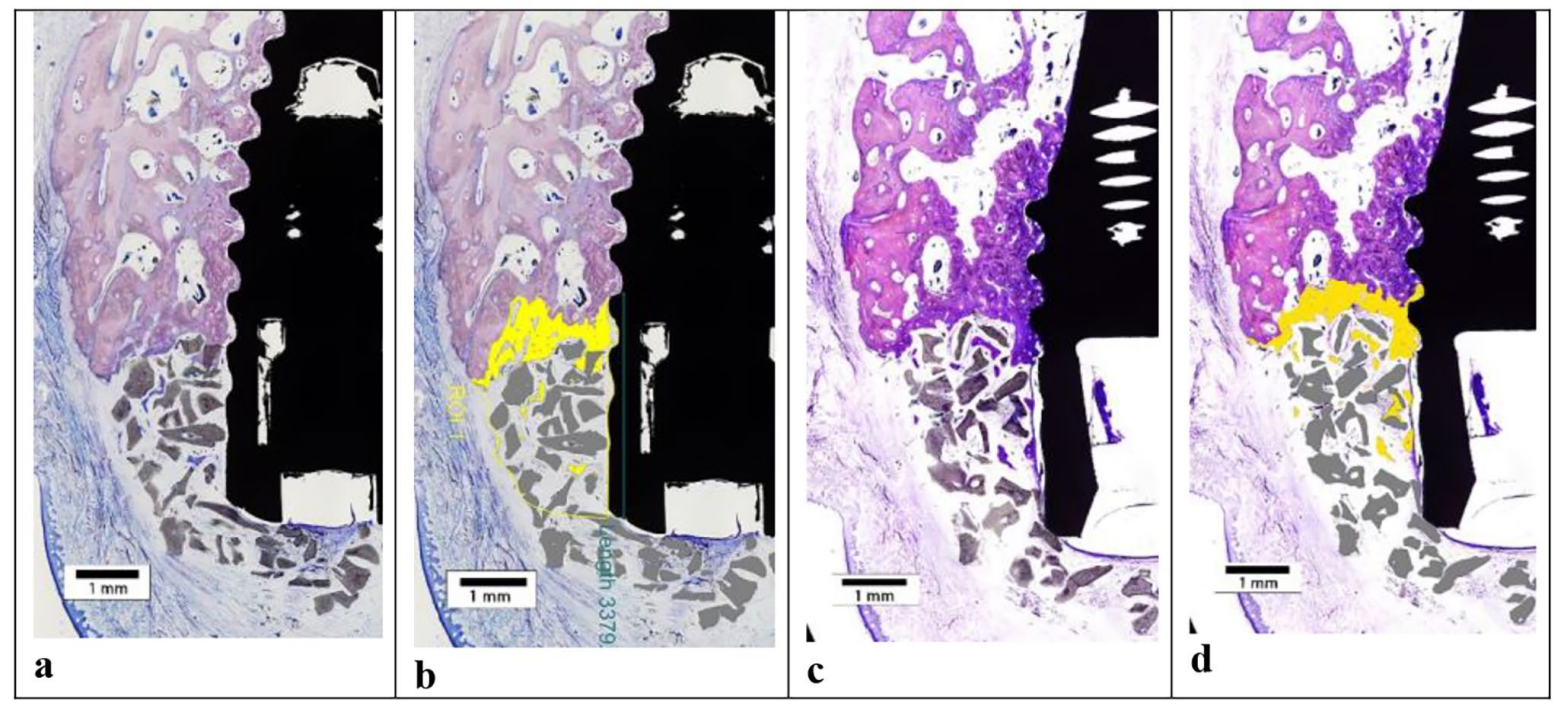
Histologic specimen from a test (membrane) site. The histological image at (**a**) the central (mid-buccal) section is shown, and (**b**) the software enhancement of the same histologic section. The histological image at a lateral section of the same specimen, showing (**c**) the original image, and (**d**) the software enhanced image. Grey represents the bone substitute particles. Yellow represents new bone ingrowth

**Fig. 4:**
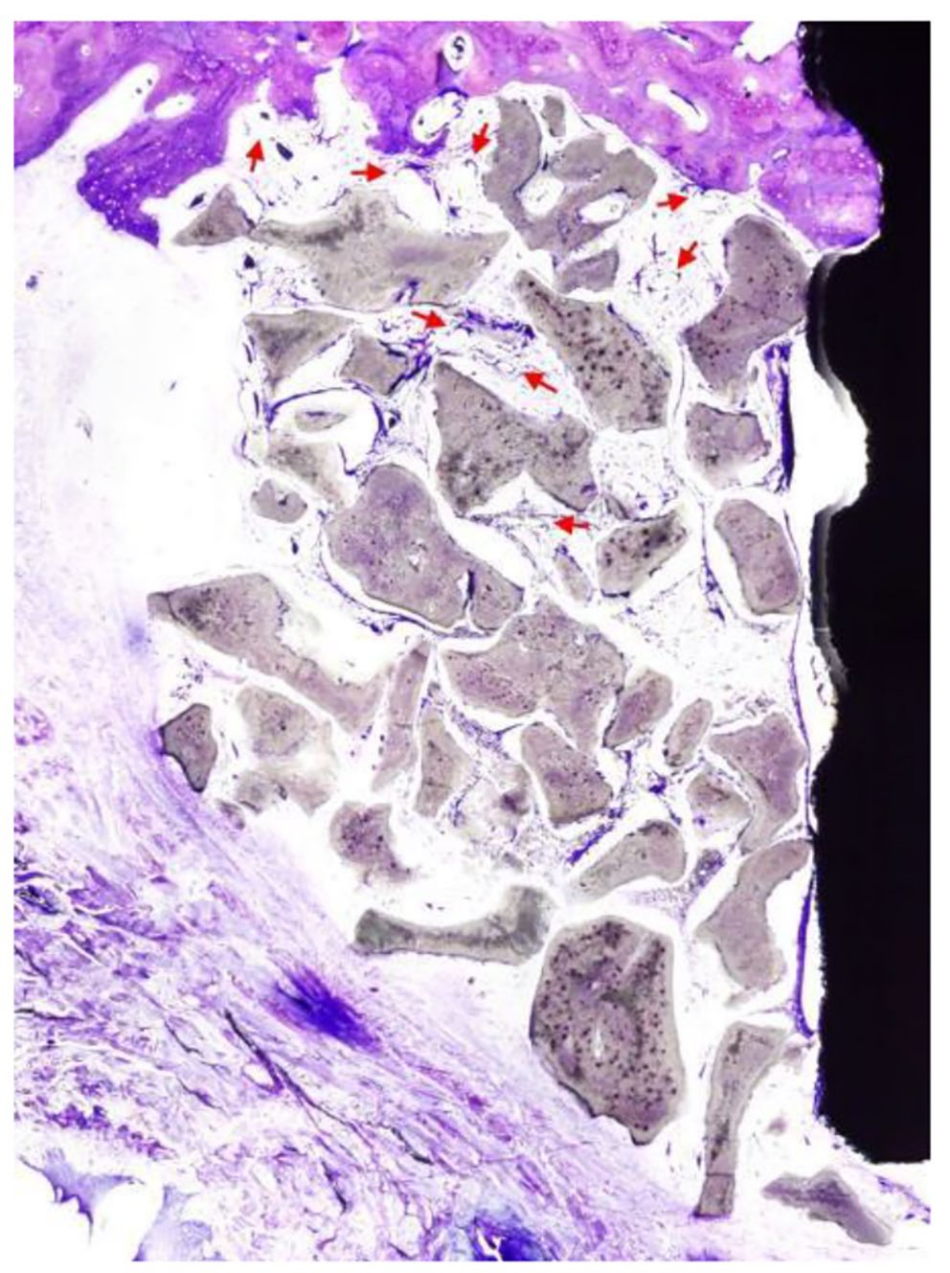
Within the grafted area, bone substitute particles were surrounded by a vascular non-inflamed connective tissue matrix (red arrows demonstrate blood vessels). Towards the outer side of the graft, this connective tissue was less vascular

Histologically, direct bone to implant contact was observed at the lingual aspect and apical to the buccal defect in all specimens, which confirmed osteointegration of the implants. At all sections, the experimental bone defects at control and test sites were similar in appearance and were completely filled coronal to the level of the implant shoulder with a new tissue comprising bone, BS and non-mineralized tissue. No remnants of membrane were observed. In general, the coronal and buccal outline of the RA resembled an intact alveolar ridge, except in one specimen where loss of graft volume in the coronal region resulted in a flattened outline. Without exception, graft material was present coronal to the implant shoulder. At central sections, newly regenerated bone was in general confined to the apical region (base of the defect), covering BS particles adjacent to the original bone (Fig. 3a, b). The histological appearance was similar between test and control sites. The new bone was comprised of woven and lamellar bone. In lateral sections, a greater proportion of new bone was observed in the apical and central regions of the RA compared to the central sections (Fig. 3c and d). At lateral sections, the histological appearance was similar between test and control sites. In two specimens (one test and one control), an intra-bony defect was present at the base indicating that bone resorption had occurred following preparation of the experimental defects. There was a greater area of new bone in these two specimens compared to the other sites which had a flat base with no intra-bony compartment. The majority of the new tissue within the defect in central sections was comprised of BS particles surrounded by a vascular non-inflamed and non-mineralised connective tissue matrix (Fig. 4). Towards the coronal and outer regions of the graft, this connective tissue was less vascularised. Osteoclastic activity was observed around BS particles nearer the base of the defect. Coronally, minimal osteoclastic activity around BS particles was seen. The fBIC was observed to be close to the base of the defect in all specimens. In several test and control sections, BS particles were observed external to the defect near the base. The particles were surrounded in a non-inflamed avascular fibrous connective tissue continuous with the connective tissue within the RA, with minimal osteoclastic activity observed.

### Histomorphometric analysis

At central sections, there was no statistically significant difference between test and control groups for S-fBIC, with a mean distance in both groups of 2.6 mm (Table 1). The fBIC was generally located close to the base of the defect indicating minimal regeneration of new bone and lack of osseointegration in the coronal region of the defect. There was no statistically significant difference between groups for buccal bone width at 1 mm, 2 mm, 3 mm, 4 mm and 5 mm increments from the implant shoulder (Table 1). The width of the RA at 1 mm from S was 1.4 to 1.5 mm, indicating the presence of a thick buccal bone wall comprised predominantly of BS. The buccal bone doubled in width between the 1 mm and 5 mm increments from S.

**Table 1:** Summary of histomorphometric measurements for S-fBIC and buccal bone thickness at 1 mm increments from S, expressed as the mean, median, minimum and maximum values for each variable, and associated *p*-values (data is for 9 paired specimens)

Variable	Membrane	Mean (± sd)	Median	Minimum	Maximum	*P* value
S-fBIC (mm)	No	2.65 ± 0.85	2.79	1.16	3.72	0.930
	Yes	2.63 ± 0.71	2.88	1.70	3.49	
Buccal bone thickness (mm)						
1 mm from S	No	1.54 ± 0.48	1.60	0.77	2.18	0.596
	Yes	1.41 ± 0.59	1.44	0.56	2.41	
2 mm from S	No	2.05 ± 0.54	1.98	1.04	2.73	0.791
	Yes	2.10 ± 0.66	2.12	1.11	3.02	
3 mm from S	No	2.45 ± 0.46	2.56	1.79	3.10	1.000
	Yes	2.47 ± 0.75	2.44	1.35	3.43	
4 mm from S	No	2.64 ± 0.66	2.83	1.59	3.37	0.596
	Yes	2.83 ± 0.63	2.75	1.62	3.51	
5 mm from S	No	2.83 ± 0.68	2.94	1.54	3.66	0.860
	Yes	2.96 ± 0.52	2.97	1.99	3.65	

S-fBIC = implant shoulder to first bone to implant contact

Two specimens (one test and one control) had deep intrabony defects at the base of the experimental defects which were substantially different from the intended flat-base defects seen in remaining samples. Since the new bone formation within the intrabony defects were likely to confound the histomorphometric analysis, these 2 specimens were excluded from the subsequent analysis of RA, BS, B, %B and MT, leaving 7 paired specimens for the analysis. At central sections, there was no statistically significant difference between groups for RA, BS, B, %B and MT (Table 2). The difference for %BS and %MT approached significance (*p* = 0.052), favouring the membrane group. These results indicate that at central sections, membrane use tended to favour an increase in %BS and %MT but not B ingrowth when compared to no membrane.

**Table 2:** Histomorphometric measurements at central and lateral sections of the variables of interest by groups expressed as the mean, median and interquartile range (data is for 7 paired specimens)

	Membrane	Central			Lateral		
		Mean ± sd	Median (interquartile range)	*p*-value	Mean ± sd	Median (interquartile range)	*p*-value
RA (mm^2^)	No	5.39 ± 1.31	5.32 (4.29, 6.46)	0.205	5.41 ± 0.70	5.23 (4.85, 6.01)	0.673
	Yes	4.25 ± 1.13	4.12 (3.35, 5.10)		5.20 ± 1.07	5.39 (4.21, 6.24)	
BS (mm^2^)	No	1.53 ± 0.51	1.73 (0.89, 1.87)	0.933	1.77 ± 0.47	1.58 (1.41, 2.40)	0.933
	Yes	1.48 ± 0.51	1.55 (1.16, 1.79)		1.68 ± 0.54	1.59 (1.30, 2.05)	
%BS	No	27.93 ± 5.76	26.79 (23.28, 35.23)	0.052	31.90 ± 5.88	31.31 (26.77, 38.06)	0.800
	Yes	34.44 ± 5.14	35.15 (30.35, 38.85)		31.29 ± 5.44	32.42 (25.78, 36.02)	
B (mm^2^)	No	0.38 ± 0.47	0.15 (0.02, 0.79)	0.933	0.65 ± 0.37	0.54 (0.38, 0.75)	0.447
	Yes	0.38 ± 0.32	0.36 (0.05, 0.52)		0.66 ± 0.12	0.66 (0.55, 0.76)	
%B	No	7.37 ± 10.26	2.08 (0.22, 12.25)	0.554	12.24 ± 6.85	10.54 (8.48, 12.75)	0.272
	Yes	8.82 ± 7.12	8.49 (1.28, 17.28)		12.76 ± 2.69	11.62 (10.78, 15.39)	
MT (mm^2^)	No	1.91 ± 0.55	1.83 (0.82, 1.89)	0.800	2.42 ± 0.49	2.10 (1.73, 2.85)	0.673
	Yes	1.86 ± 0.61	1.26 (1.20, 1.65)		2.33 ± 0.56	2.01 (1.50, 2.31)	
%MT	No	35.30 ± 7.97	35.45 (31.58, 39.04)	0.052	44.43 ± 5.72	43.33 (39.03, 50.67)	0.800
	Yes	43.26 ± 4.58	43.74 (40.12, 47.53)		44.77 ± 4.15	44.62 (42.13, 47.71)	

RA - regenerated area

BS - bone substitute area

B - new bone area

MT = mineralized tissue (new bone + bone substitute) area

The percentages were related to the Regeneration Area

At lateral sections, there was no difference between groups for RA, BS, %BS, B, %B, MT and %MT (Table 2). This indicates that membrane use had no influence on these parameters.

Since there were no differences in central and lateral sections for all parameters except %MT in central sections, the groups were combined to analyse differences between central and lateral sections. No statistically significant differences were observed between central and lateral sections for RA, BS and %BS (Table 3). However, there was statistically more B, %B and MT at lateral sections compared to central sections. The difference in %MT between groups approached significance.

**Table 3:** Histomorphometric measurements comparing central and lateral sections of the variables of interest by groups expressed as the mean, median and interquartile range (data is for 7 paired specimens)

	Central		Lateral		
	Mean ± sd	Median (interquartile range)	Mean ± sd	Median (interquartile range)	*p*-value
RA (mm^2^)	4.82 ± 1.32	5.88 (4.79, 7.40)	5.31 ± 0.88	5.31 (4.63, 6.11)	0.209
BS (mm^2^)	1.51 ± 0.49	1.57 (1.09, 1.81)	1.72 ± 0.48	1.59 (1.41, 2.14)	0.367
%BS	31.18 ± 6.24	32.48 (24.92, 36.11)	31.60 ± 5.45	36.53 (31.38, 40.59)	0.556
B (mm^2^)	0.38 ± 0.38	0.33 (0.05, 0.59)	0.65 ± 0.26	0.63 (0.51, 0.75)	0.021
%B	8.09 ± 8.52	6.61 (1.14, 13.51)	12.50 ± 4.83	10.78 (10.38, 13.57)	0.017
MT (mm^2^)	1.88 ± 0.56	1.86 (1.43, 2.38)	2.37 ± 0.51	2.27 (2.04, 2.85)	0.025
%MT	39.28 ± 7.44	39.58 (34.54, 44.77)	44.60 ± 4.80	43.78 (41.15, 48.45)	0.055

RA - regenerated area

BS - bone substitute area

B - new bone area

MT = mineralized tissue (new bone + bone substitute) area

The percentages were related to the Regeneration Area

## Discussion

The buccal dehiscence defect is a challenging peri-implant defect for bone regeneration procedures. In the present study, a regenerated bone wall comprised of BS, B and non-mineralised tissue consistently formed. However, there was a difference in the proportion of B within the sections. At central sections, there was a tendency for more %MT when a membrane was used. The increased %MT was due to the presence of more BS throughout the RA but not more B ingrowth. This suggests that membrane use retains more BS, but does not influence B ingrowth into the graft in central sections. Thus, the clinical concept that a membrane helps to stabilise and maintain the graft volume within a defect [2] is supported by these findings.

In contrast, there was more B, %B and MT at lateral sections compared to central sections, which confirms that bone ingrowth in dehiscence defects occurs mainly from the proximal bone walls rather than from the central base of the defect. The defects created were 5 mm in height and 3 mm in width at the base. Thus, a greater surface area of bone was present at lateral surfaces rather than at the base. In a canine mandibular defect model, Schenk and co-workers showed that new bone formation in membrane protected defects occurred from the cut surface of the defects [15]. This is supported by a more recent study from Park et al. where they used a posterior mandibular defect to study the outcomes of transmucosal healing versus submerged healing with or without GBR involving graft and membrane. In both groups without GBR they reported spontaneous new bone formation along the walls and floor of the defects. The sites with GBR had well integrated particles within the newly formed bone, but in this study the healing period was 5 months [16]. The cut surfaces exposed the trabecular compartment from which new blood vessels were derived. In bone healing, angiogenesis is a precursor to osteogenesis [17]. In a pre-clinical study, it was demonstrated that a significant vascular supply to the buccal bone plate originates from the proximal bone walls [18]. These findings may explain the clinical observation that wider dehiscence defects have reduced bone regenerative potential [19] and greater risk of mucosal recession when compared to narrower dehiscence defects [20].

The findings of this study are largely in agreement with that of Janner and co-workers [10]. In this study in a canine model, maxillary second incisors and first premolars were extracted and implants placed five weeks later. Standardised dehiscence defects were created on the buccal aspect of the implants, and were randomly allocated to the following treatment groups: DBBM + membrane (group D + M), autogenous bone chips + DBBM + membrane (group A + D + M), DBBM alone (group D) or autogenous bone chips + DBBM (group A + D). At three weeks, there were no differences between groups D + M, D and A + D for new bone ingrowth into the grafted area, which was similar to the findings of the present study. Group A + D + M show significantly more new bone ingrowth due to the incorporation of a layer of autogenous bone chips against the exposed implant surface. At 12 weeks however, groups with membrane (D + M and A + D + M) showed significantly more bone ingrowth. This contrasts with the findings of the present study which showed that after 6 weeks, there was no effect of membrane use except on %BS and %MT. The difference may be due to the longer healing (12 weeks versus 6 weeks in the present study) which may have allowed more time for bone ingrowth. In this regard, the observation that BS particles were surrounded by a vascular and non-inflamed connective tissue is interesting. It may be speculated that these conditions may be conducive for bone ingrowth if left undisturbed over time. Previous studies have shown that new bone regeneration within membrane protected defects is time dependent [21, 22].

It is possible that the membranes may have moved on closure. A closure technique was used that minimised disruption of the membrane and so limited any movement. Pins and tacks are not routinely used with membranes so the technique in this study mirrors clinical practice. The histomorphometric dimensions of the defects compared to that measured clinically were different. However, the defects were all similar sizes. The discrepancy maybe due to the anatomy of the maxilla and application of the probe to measure the defects resulting in an over-estimation.

## Conclusions

Under the conditions of the study, the use of a membrane had a positive effect on retention of bone substitute particles grafted into the experimental defect, but had no effect on new bone ingrowth into the graft. Lateral sections showed significantly more bone ingrowth, percentage new bone and mineralized tissue when compared to central sections, confirming that new bone ingrowth takes place mainly from the lateral bone walls of the experimental dehiscence defect.
